# Solving the puzzle of neurological diseases: an interview with Huda Zoghbi

**DOI:** 10.1242/dmm.029751

**Published:** 2017-05-01

**Authors:** Huda Y. Zoghbi

## Abstract

Huda Zoghbi's achievements in the field of neurology are internationally acclaimed. She is best known for elucidating the genetic basis of two complex neurological disorders, spinocerebellar ataxia type 1 and Rett syndrome, and has been honored with many prizes, including The Shaw Prize in Life Science and Medicine in 2016 and the 2017 Breakthrough Prize for Life Sciences. A diligent and creative research scientist at the bench, a respected lab mentor and founding Director of the Jan and Dan Duncan Neurological Research Institute at Texas Children's Hospital, her inspiration has always been the burning need to help patients faced with devastating neurological problems. Her pursuit of the mechanisms mediating spinocerebellar ataxia and Rett syndrome has been dogged, requiring 30 years of focused effort. As highlighted in this interview, her work is now paying dividends by starting to reveal potential therapeutic targets for these intractable and complex disorders.

## Abstract (precis)

**Summary:** Huda Zoghbi discusses her remarkable journey from the clinic to the bench, highlighting the experiences and collaborations that inspired her interest in neurological diseases such as spinocerebellar ataxia type 1 (SCA1) and Rett syndrome.

**Figure:**
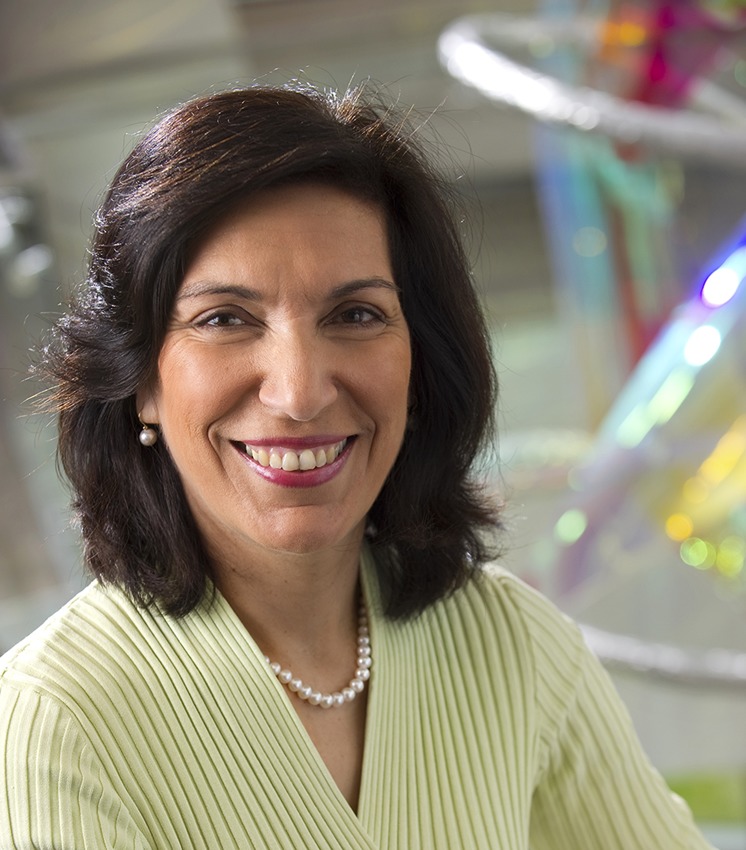
Photo credit: Paul V. Kuntz

Huda Zoghbi is originally from Lebanon and completed her Biology degree at the American University in Beirut in 1975. She was part way through her first year at medical school when Civil War erupted. A move to the USA for the summer became permanent when the situation at home worsened, and she enrolled at Meharry Medical College in Nashville, graduating in medicine in 1979. During her clinical residency at Baylor College of Medicine in the early 1980s, her pain and frustration at not being able to offer any explanation or treatment to parents of children with complex neurological conditions set her on the path to become a research geneticist. A postdoctoral research fellowship, in Arthur Beaudet's lab at the Institute of Molecular Genetics at Baylor, marked the beginning of multi-year search for the genetic basis of two rare conditions: spinocerebellar ataxia type 1 (SCA1) and Rett syndrome. In the competitive and ever-evolving arena of genetic research, Huda has worked in long-term collaboration with Hugo Bellen, Juan Botas and most notably with Harry Orr at the University of Minnesota, with whom she jointly discovered the causative gene for spinocerebellar ataxia type 1. Still at Baylor, Huda currently holds professorships in Pediatrics, Neurology, Neuroscience, and Molecular and Human Genetics. She is a Founding Editor of Disease Models & Mechanisms (DMM).

**Tell us a little about your early life: did you always want to be a doctor?**

I grew up in a family that encouraged learning. I was, and still am, enchanted by Shakespeare, Wordsworth and Jane Austen, and when I was around 16, I really wanted to be a writer. Biology certainly did interest me, but it was actually my mother's suggestion to consider medicine. She saw that I was good at science and she felt that it would be a better career for me. She was right; she knew me better than I knew myself. I still love literature and feel that this passion comes across in my scientific writing. My research papers tell a story and people really relate to that.

“I still love literature and feel that this passion comes across in my scientific writing. My research papers tell a story and people really relate to that”

**You trained as a pediatric clinician and intended to specialize in cardiology. Why the switch to neurology?**

I arrived at Baylor planning to specialize in pediatric cardiology, but I quickly became fascinated by the complex problems I encountered during my neurology rotation. Every case was an intricate puzzle that required its own special solution. As a neurologist, the more experience you have mapping symptoms to specific brain regions, the better you are at solving the puzzle. A thorough history is a key part of the process; just talking to the child and the parents can give you an amazing amount of information. Often, I found that a physical examination and further tests were valuable for confirming what I'd already deduced from learning the patient's history.

I so enjoyed this process that I couldn't leave neurology behind. As I continued to see patients, however, it hit me just how devastating these problems are for the families involved. It was so painful to have to explain to parents that their child's problem was likely to be genetic, so future children could also be affected, but then to have to tell them that we didn't know the underlying cause or how to fix it. I became a mother around this time too, which brought into focus just how much distress these families were experiencing. I was inspired by the clinical cases I saw and realized that the best way I could help would be to commit to being a scientist and to investigate the causes of these neurological disorders.

“As a neurologist, the more experience you have mapping symptoms to specific brain regions, the better you are at solving the puzzle”

**How important was your postdoctoral fellowship in Art Beaudet's lab in shaping your research interests when you later set up your own lab?**

When I started in Art's lab, he was working on urea cycle defects and metabolic disorders caused by specific enzyme deficiencies, while also attempting to clone the cystic fibrosis gene. I admitted to him that none of these projects interested me and that I wanted to pursue more complex problems such as Rett syndrome. Even though I knew nothing about molecular biology he was still willing to take me on and to support me, but told me very firmly that I couldn't start with Rett. He advised that I learn the ropes of molecular biology with an obvious Mendelian problem. Late-onset neurological disorders really interested me and we knew of a large family in the Houston area affected by spinocerebellar ataxia type 1 (SCA1), so that's what I chose to study.

Art was a superb mentor. He was so accommodating and generous but he also knew when he had to be firm and steer me away from risky projects! He also taught me the necessity of showing scientific rigor and for being ‘feisty’ when presenting or writing about research outcomes. He continued to support me long after I started my own lab and we would meet regularly to exchange ideas – we became good friends and continue to be so.

**After setting up your own lab, how did you reach out to Harry Orr and begin collaborating to uncover the genetic basis of SCA1?**

It is a unique and sweet story. SCA1 is rare; it affects only one in 100,000 people. As I began work with the Houston family in 1988, Harry Orr was already working on samples from a large Minnesota family. Both of us mapped the disease to chromosome 6, but to two different regions. Great, I thought, we are working on two different diseases. When I generated radiation hybrids for chromosome 6, I called Harry and offered to share them and to collaborate. As our work progressed, I realized while mapping the gene using a new marker that we were both actually working on the same gene. When I called Harry to break the news, the first thing he said was, ‘Do you want your radiation hybrids back?’. I said ‘No’ and suggested we work together, and that's when he paused for a few seconds because it's a big decision. This was in the era of positional cloning when cloning a disease gene was a challenge – it was quite a feat and very competitive. He had to think about what it might mean for the other fellows working in the lab, but after those few seconds, he agreed. We pooled our resources and moved faster than if we had worked independently. It was a special moment when we both discovered the mutation that revealed the genetic basis of the disease on the same day in April 1993. We still share all of our results and our plans and talk endlessly about the best way forward in our research and we're the best of friends. My husband threw a party for the Breakthrough prize and the biggest surprise was Harry walking into the house to be with us. Collaborations can provide a long-term means to maximize resources, and I hope that science will become more and more collaborative.

**What did the discovery of the SCA1 gene, ataxin-1, mean in terms of understanding the mechanism of disease?**

It took a while to figure this out. After the gene discovery in 1993, it took a lot of genetic work in Harry's and my lab over the next 20 years to understand the mechanism. Two key findings came from animal models where our results kept showing that the disease develops when ataxin-1 gains rather than loses function – but we didn't know how.

Using animal models, we were able to look at the biochemistry and genetic interactions and finally showed that the polyglutamine expansion (polyQ) alters interactions with other cellular proteins. The underlying disease mechanism involves a gain of native function and a partial loss of function, but it is the gain of normal function that drives degeneration. I believe this is an emerging theme in neurodegeneration; many proteins are involved and their levels and function have to be ‘just right’, otherwise the brain – particularly the ageing brain – becomes vulnerable.

We then did genetic studies in which we lowered the levels of the ataxin-1 protein by 20% and, lo and behold, we significantly rescued many disease symptoms. Our high-risk strategy was then to search for factors that, when inhibited, lower ataxin levels, so that we could hopefully identify a therapeutic target. We worked on it for three years, together with Juan Botas who brought in the *Drosophila* model of SCA1, and just when I was losing hope, we ended up with a reliable screen that worked and we had our first druggable target.

**During this time, why did you continue to be interested in uncovering the genetic basis of Rett syndrome? What makes this disease so devastating?**

Although my main work in those early years focused on ataxia, the clinical course of the disease in Rett syndrome inspired me to continue working on it ‘on the side’. It is almost a choreographed disease course and that is what struck me. Rett is vastly different from most neurological diseases, which fall into one of two major categories. The classic neurodevelopmental disorders, characterized by a physical brain defect, cause symptoms from birth; then degenerative disorders, such as lysosomal storage disorders, cause the child to lose milestones gradually due to degenerative changes that are life-limiting. Rett syndrome girls are born without obvious problems. They smile, walk, start to say a few words, they are social and start to use their hands. That stops suddenly, often around the age of 18 months, but there is no neuronal degeneration to explain the change. The timing of onset varies, but most girls affected show symptoms by the age of two. Deterioration rapidly follows; the girls start wringing their hands and over a period of 5 to 7 years, every few months will bring on another symptom – balance problems and tremulousness, breathing problems (including apnea and hyperventilation), seizures, dystonia, stiffness in the arms, and digestive system malfunction. They go on to live into adulthood still showing no degeneration to explain these devastating effects. This is why I figured anything that is so choreographed has to be caused by a gene and I was determined to find the gene.

“The clinical course of the disease in Rett syndrome inspired me to continue working on it ‘on the side’. It is almost a choreographed disease course and that is what struck me”

Rett typically arises spontaneously in a family, and in the 1980s and 1990s we didn't have the technology to clone and map any gene responsible for a sporadic genetic disorder. As it only affects girls, we started to collect evidence on the X-chromosome and I studied one family where the mother had daughters from two different spouses. Both had the disease and I rationalized that non-random X inactivation was at play in the mother, which would favor the healthy X-chromosome and make her asymptomatic, while she would still be able to pass the affected X-chromosome to her daughters. I demonstrated 100% skewing, but in the early 90s I couldn't publish the paper in which I reported this – none of the major genetic and scientific journals would take it. Eventually, I published it in a small journal. I kept on going, working on Rett while still focusing on ataxia, and the path was laborious and littered with red herrings. None of the technologies we rely on now were in place back then and it took typically 6 to 12 months of work to eliminate a gene. Being systematic was the best approach and, eventually, we did show that the genetic cause of Rett syndrome is loss-of-function mutations in the gene encoding methyl-CpG binding protein 2 (*MECP2*). It was exactly 16 years from the day I saw my first Rett patient to the day the gene was cloned. The discovery provided evidence that a sporadic intellectual disease/autism-spectrum disorder, can be caused by a genetic defect.

“It was exactly 16 years from the day I saw my first Rett patient to the day the gene was cloned”

**What do we now know about *MECP2* and what are the key gaps in our knowledge of this gene?**

Adrian Bird showed that the MeCP2 protein binds methyl CpG, and recent studies from many labs, including ours, have demonstrated that it also binds to non-CG methylation, particularly CA. We still have a lot to learn, but what I can say with confidence is that the protein is essential for the function of many cells in the brain – many neurons are compromised in its absence. From a neurobiology standpoint, this is a useful feature as cell-specific *Mecp2* knockout mice can be used to study the function of different cell types. We also know that the amount of protein present is crucial; in males, the gene mutation is lethal because it means they cannot produce MeCP2 at all. Females with a mutation in *MECP2* have an absent or abnormal protein in half of their cells and normal protein in the other half, resulting in Rett syndrome. Too much protein is also harmful; mice with an extra copy of *Mecp2* show progressive neurological damage and quite a few people with this duplication have now been identified. *MECP2* triplication syndrome, which is more severe, occurs in people with three copies of the *MECP2* gene. The level of this protein is finely tuned in the brain. SCA1 and Rett have both shown me the importance of protein levels. I really think that the brain adheres to the ‘Goldilocks principle’ when it comes to protein levels – they have to be exactly right.

**How far are we from being able to use this knowledge to restore *MECP2* function in Rett syndrome?**

Adrian Bird has shown that it is possible to reverse Rett syndrome in adult mice, and we now need to ask if can we do it in humans: whether pharmaceutically, by gene therapy or by neuronal network modulation. Gene therapy approaches are feasible if we can really show that they would work for mature brain disorders; some investigators are pursuing this. Our studies (with Jianrong Tang) on deep brain stimulation of the hippocampus in animal models have had promising effects on learning and memory and we are testing it now for its potential to help with motor problems.

For the duplication disorder, we have taken the strategy of using antisense oligonucleotides (ASOs), with Ionis, to normalize the level of MeCP2. We've shown that in adult mature mice, protein levels can be normalized and disease symptoms reversed. This is very encouraging because ASOs have been used successfully and safely in children. We must, however, do more work to show we can control the reduction of MeCP2 – too little will cause Rett-like problems.

**Other than Rett syndrome, have you pursued other curiosity-driven projects?**

Yes. I was quite impressed by how much we were learning about nervous system development from *Drosophila* studies. So I turned to my colleague, Hugo Bellen, and asked his advice about an important gene to study in mammals. He guided me to the atonal gene discovered by the Jan lab as being essential for development of proprioreceptors and the hearing organ in the fruit fly. I isolated the mouse homolog (*Math1/Atoh1*) and made animal models that revealed many exciting and unanticipated results.

The Atoh1 protein is a transcription factor that specifies the development of particular types of neurons. I learned so much real developmental biology from studying this gene but more than that, our studies revealed that the gene has a greater impact on human health than I could ever have imagined. It turns out to be important for inner ear hair cells in the cochlea and vestibular systems, for cerebellar granule neurons, Merkel cells, and intestinal secretory cells. We are now studying it in the context of breathing. We learned that loss of this protein makes some neurons more sensitive to carbon dioxide, making it relevant to sudden infant death syndrome and congenital breathing disorders. It's incredible just how much the *atonal* gene in *Drosophila* has taught us about mammalian (including human) developmental biology, and its relevance to diseases ranging from deafness to cancer to breathing.

**Animal models have clearly been so important in your work. What are the key advantages and limitations of animals as models of neurological diseases?**

This is a very important question; papers have been published using animal models that do not replicate in people for various reasons. Sometimes the models don't have construct validity and this is common in neurodegeneration. Diseases such as Alzheimer's and Parkinson's, including the rare forms that are caused by a single gene mutation, are difficult to reproduce in a mouse model because the animals simply don't live long enough. In the case of polyQ diseases, we are fortunate because we can simply extend the polyQ length to enable the phenotype to appear in the short lifespan in the mouse. This is why I trust the animal models that we have used to study SCA1.

“Diseases such as Alzheimer's and Parkinson's, including the rare forms that are caused by a single gene mutation, are difficult to reproduce in a mouse model because the animals simply don't live long enough”

The Rett mouse model has construct validity and reproduces all the features of the human disease. The question is what will we learn from these mice – is it going to be translatable to humans? Harry Orr showed many years ago that mice that overexpressed *ATXN1* in Purkinje cells, developed ataxia and Purkinje cell degeneration. He then turned the gene off at 3 months, 4 months and 6 months and found that while reversal happened at the first two time points, after 6 months it was partial because cells had been lost. Will we see the same reversal with therapeutics in people? On one hand I think we shouldn't assume that because we can take a massively symptomatic mouse and reverse its symptoms, we are going to be able to do the same in a massively symptomatic human. On the other hand, I feel we should also do our best to start treatments and trials as early as possible in the disease course to have the best chance to reverse symptoms and make a difference to the disease process.

As a neurologist, I have been really impressed by the power of *Drosophila* for informing human disease research, and my collaborations with Hugo Bellen and Juan Botas have been illuminating. Zebrafish are also useful for functional studies that are more difficult and time-consuming in mice. If I don't understand what a particular gene does in a mammal but I can find its homolog in *Drosophila*, then I can study it more effectively in the fruit fly, dissect the pathway, and then go back into a mammalian model and validate the findings. With so many genomes and exomes being sequenced nowadays, there are so many variants to study functionally and determine if they are consequential. Cross-species studies can work beautifully to unravel function and the impact of variants on the function in a relatively short period of time, and they can also save money.

**What are you currently most excited about in the lab?**

After 25 years I feel I finally understand the biology of the disorders I am studying well enough to start developing therapeutics, which is really exciting. I've mentioned some examples already, and we've also recently identified a new target for tau, one of the proteins that drives neurodegeneration in Alzheimer's. The target is an enzyme that you can inhibit to reduce the level of tau, and this rescues many of the symptoms in a tauopathy model. This target had not been discovered before, which is remarkable when you think of the vast amount of work that has been done on tau. I think it's because we used unbiased cross-species screens that allowed the biology to inform, rather than looking directly for a therapeutic target. Understanding the biology can reap such rewards. I am excited about using this strategy to gain more insight into the biology of proteins such as MeCP2 and ataxin-1. Unravelling the biology of these proteins – what modifies them post-translationally, what controls their levels, what controls their complex formation – will expand our knowledge while also yielding potential therapeutic targets. I am realistic, and humbled by the challenges of developing a small molecule for therapy. But I think the more candidates we have, the more we increase the chances of one day being able to do something for people affected by these diseases.

**Nowadays, do you have much interaction with patients?**

Less than I used to. I no longer provide their clinical care but I now enjoy interacting with them socially. They come to my office and often visit the lab. I make every effort to keep in touch and keep them up-to-date with my research. It's still an important aspect of my work.

**What advice do you give to early-career scientists?**

First of all, you have to pick a problem you are curious and passionate about rather than chase the funding or trends. When you're excited about something you will stick with it, even when you have to go through years of getting negative data. Second, you need to be highly focused at the start of your research career. Focus allows you to be the expert on the problem, you develop deep knowledge, you ask creative questions, and you cannot help but make an impact. Thirdly, I would advise working at the bench as long as possible. When I started I was in the lab at the bench all the time, not on the computer and not traveling extensively to meetings. People travel a lot more these days, and I think that has an impact on the time they can spend at the bench.

“After 25 years I feel I finally understand the biology of the disorders I am studying well enough to start developing therapeutics, which is really exciting”

**You were recently awarded the Breakthrough Prize in Life Sciences (congratulations!), in recognition of your unique contributions to understanding neurological diseases. What was your reaction when you found out about the award?**

Cori Bargmann called me on my cell to tell me about the award – I was stunned and I think I froze. I was very honored and touched and immediately thought of everyone in my life who made this possible; my mentors, Art in particular, my trainees who contributed to this prize, my colleagues and collaborators. I was really nervous about the ceremony; Yuri Milner (one of the co-founders of the prize) had told me it was like the Oscars for science. Although I really don't like that kind of attention, now I see the value of glamorizing science and discovery. I can say the prize captured the attention of the public and I've since had so many emails from young people, particularly females, saying they were excited and wanted to become a scientist! The impact on young people, particularly women, was so rewarding.

A touching moment at the actual awards ceremony was when Sergey Brin presented me with the prize saying, ‘I give this to you, from a Russian immigrant to a Lebanese immigrant’. That really resonated with the immigrant community, both Arab-American and Lebanese-American. I got really touching emails from people feeling validated, hopeful and proud. The monetary aspect of the award is tangible and can be used to do a lot of good in science and society, but these more non-tangible benefits that I hadn't expected.

**What do you enjoy doing outside of the lab?**

I love opera and hiking and exercising. I'm also a gourmet cook who loves to eat! And, of course, I still find time to read.

**If you hadn't chosen science, what would you be doing now?**

Something in literature, definitely.

